# Crystallographic characterization of the high-potential iron-sulfur protein in the oxidized state at 0.8 Å resolution

**DOI:** 10.1371/journal.pone.0178183

**Published:** 2017-05-22

**Authors:** Hiraku Ohno, Kazuki Takeda, Satomi Niwa, Tomotaka Tsujinaka, Yuya Hanazono, Yu Hirano, Kunio Miki

**Affiliations:** Department of Chemistry, Graduate School of Science, Kyoto University, Sakyo-ku, Kyoto, Japan; Universidade Nova de Lisboa, PORTUGAL

## Abstract

High-potential iron-sulfur protein (HiPIP) is a soluble electron carrier protein of photosynthetic bacteria with an Fe_4_S_4_ cluster. Although structural changes accompanying the electron transfer are important for understanding of the functional mechanism, the changes have not been clarified in sufficient detail. We previously reported the high-resolution crystal structures of HiPIP from a thermophilic purple bacterium *Thermochromatium tepidum* in the reduced state. In order to perform a detailed comparison between the structures in different redox states, the oxidized structure should also be revealed at high resolution. Therefore, in the present study we performed a crystallographic analysis of oxidized HiPIP and a structural comparison with the reduced form at a high resolution of 0.8 Å. The comparison highlighted small but significant contraction in the iron-sulfur cluster. The changes in Fe-S bond lengths were similar to that predicted by theoretical calculation, although some discrepancies were also found. Almost distances between the sulfur atoms of the iron-sulfur cluster and the protein environment are elongated upon the oxidation. Positional changes of hydrogen atoms in the protein environment, such as on the amide-hydrogen of Cys75 in the proximity of the iron-sulfur cluster, were also observed in the accurate analyses. None of the water molecules exhibited significant changes in position or anisotropy of atomic displacement parameter between the two states, while the orientations of some water molecules were different.

## Introduction

High-potential iron-sulfur protein (HiPIP) is a small (~10 kDa) soluble protein functioning as an electron carrier in photosynthetic Gram-negative bacteria [1,2]. HiPIP supplies an electron from the cytochrome *bc*_1_ complex to the photosynthetic reaction center (RC) in order to re-reduce a special pair of chlorophyll molecules in the RC. The protein possesses one Fe_4_S_4_ cluster at the molecular center. The cluster changes its redox state between [Fe_4_S_4_]^2+^ and [Fe_4_S_4_]^3+^ [3,4]. The redox midpoint potential is approximately +300 mV, and therefore the reduced form is the resting state, and is stable even under normal atmospheric conditions. In previous studies, several HiPIP homologues in the reduced state have been determined by X-ray crystallography [5–10]. The crystal structures of HiPIP from a mesophilic purple bacterium *Allochromatium vinosum* were solved both in the reduced and oxidized states at 2.0 and 1.2 Å resolutions, respectively [11,12]. In addition, the redox changes were investigated with the nuclear magnetic resonance (NMR) spectroscopy [13]. However, the changes of the bond lengths in the cluster were as small as the uncertainties of the analyses in these studies. Even more unfortunately, the coordinates and structural factors for the oxidized HiPIP are not available in the Protein Data Bank (www.pdb.org/pdb).

Details of structural changes involved in the redox reaction are indispensable for investigation of the functional mechanism of HiPIP, and thus the measurement and analysis should be performed carefully. We have performed high-resolution crystallographic investigations of HiPIP from a thermophilic purple bacterium *Thermochromatium tepidum* [14–17]. In addition, we recently succeeded in performing a charge-density analysis of HiPIP in the reduced state at 0.48 Å resolution [18]. In order to reveal detailed structural differences between the reduced and oxidized states, a high-resolution determination of the structure in the oxidized HiPIP is indispensable.

Here, we report the preparation and X-ray crystallographic analysis of the oxidized HiPIP at 0.8 Å resolution. The comparison between oxidized and reduced structures highlighted small but significant changes on bond lengths in the iron-sulfur cluster, distances between the iron-sulfur cluster and the protein environment, and the orientations of some bound waters. Our results provide unprecedented details of the structural changes between the redox reaction and the reduced state.

## Materials and methods

### Purification

HiPIP was extracted from cells of *T*. *tepidum* and purified as reported previously [14,17]. Oxidation of HiPIP was done by adding of 10 mM K_3_[Fe(CN)_6_] just before use, because the purified sample was in the reduced state. The reagent was removed by size exclusion chromatography with a Superdex G-75 column (GE Healthcare). The sample in the reduced state was prepared by adding 10 mM dithiothreitol (DTT) in order to suppress by oxidation with O_2_ during storage for a few days at 277 K. DTT was removed by size exclusion chromatography in the same way just before use.

### Spectroscopic measurements

The UV-visible spectra of the samples (1.0 mg·mL^-1^) without redox reagents were measured in 20 mM Tris-HCl buffer (pH 7.3) with a V-630 spectrometer (JASCO) in a range from 250 to 700 nm at a room temperature of ~298 K. In order to investigate the redox changes of the samples under various conditions, 100 μL solutions of oxidized HiPIP in 100 mM sodium citrate (pH 4.5) or in 1.0 M ammonium sulfate, 100 mM sodium citrate (pH 4.5) and 2 mM K_3_[Fe(CN)_6_] were incubated in 0.5 mL microtubes at 298 K, and sampled for 60 days. Absorption spectra were measured using an ND-1000 spectrometer (NanoDrop Technologies, Inc.) in a range from 220 to 750 nm at a room temperature of ~298 K. The spectra for the reduced HiPIP in 100 mM sodium citrate (pH 4.5) were also measured in the same way.

### Crystallographic methods

Crystallization experiments were performed manually by the hanging drop vapor diffusion method at 293 K using COMBOPLATE^™^ 24 WELL (Greiner). 1.5 μL of the protein solution (20 mg·mL^-1^) was mixed with 1.5 μL of a reservoir solution containing 1.5–1.6 M ammonium sulfate and 100 mM sodium citrate (pH 4.5) with or without 10 mM K_3_[Fe(CN)_6_] for the oxidized or reduced state of HiPIP. The drops were equilibrated against 500 μL of a reservoir solution and crystals grew in 2 weeks.

Diffraction experiments were performed at the beamline BL41XU of SPring-8 (Harima, Japan) (Table 1). A crystal with approximate dimensions of 1.0×0.2×0.1 mm^3^ was picked up with a nylon loop and cryo-cooled with a helium gas flow at 40 K, after soaking in a cryo-solution additionally containing 25% (*v/v*) glycerol. The root of the nylon loop was reinforced by glue in order to suppress flapping by the gas flow. The wavelength of incident X-rays was 0.40 Å and the beam size was set to 30 × 30 μm^2^. An aluminum attenuator (8.0 mm in thickness) was used to reduce X-rays to 2.9×10^10^ photons·sec^-1^, which is ~1/10 of the initial flux (3.0×10^11^ photons·sec^-1^). The data sets were collected using an MX225HE CCD detector (Rayonix). The crystal-to-detector distance was set to 250 mm. 360 frames of diffraction images were collected for each data set with an oscillation range of 0.5° and an exposure time of 0.5 sec per frame. Even strong diffractions at low resolutions were not saturated under these measurement conditions. Diffraction images were integrated with the XDS program [19] and merged with the SCALA program [20]. The absorption dose was estimated with the RADDOSE program [21].

**Table 1:** Data collection and crystallographic statistics.

Data set	Oxidized HiPIP	Reduced HiPIP
**Data collection**		
Wavelength (Å)	0.40	0.40
Temperature (K)	40	40
Oscillation range (° frame^-1^)	0.5	0.5
No. of total frames	360	360
Dose per frame (Gy)	2.0×10^3^	2.0×10^3^
Total dose (Gy)	7.3×10^5^	7.3×10^5^
**Crystallographic data**		
Space group	*P*2_1_2_1_2_1_	*P*2_1_2_1_2_1_
Cell parameters *a* (Å)	46.336	46.330
*b* (Å)	58.813	58.811
*c* (Å)	23.438	23.423
Resolution range (Å)	15–0.8 (0.84–0.80)	15–0.8 (0.84–0.80)
No. of reflections (total/unique)	399,279/65,319	399,394/64,920
Redundancy	6.1 (2.9)	6.2 (3.0)
Completeness (%)	95.3 (74.7)	95.2 (75.2)
*I/σ*(*I*)	9.2 (1.7)	8.7 (1.8)
Wilson *B* (Å^2^)	2.9	3.3
*R*_sym_^a^ (%)	11.3 (53.3)	11.5 (49.3)
*R*_p.i.m._^b^ (%)	5.3 (39.4)	5.4 (39.4)
CC_1/2_ (%)	99.7 (71.0)	99.6 (71.8)
*R*_iso_^c^ (%)	15.9 (53.3)	-

Values in parentheses refer to the highest resolution shell.

^a^ *R*_sym_ = Σ_hkl_Σ_i_| *I*_hkl,i_−<*I*_hkl_>|/Σ_hkl_Σ_i_ *I*_hkl,i_.

^b^ *R*_p.i.m._ = Σ_hkl_ [1/(n_hkl_-1)]^1/2^Σ_i_| *I*_hkl,i_−<*I*_hkl_>|/Σ_hkl_Σ_i_ *I*_hkl,i_. Bijvoet pairs of the data were kept separate but were scaled simultaneously, and *R*_sym_ and *R*_p.i.m._ values were calculated with the merged Bijvoet pairs.

^c^ *R*_iso_ = Σ_hkl_|*I*_hkl_^Ox^−*I*_hkl_^Red^|/Σ_hkl_*I*_hkl,i_^Red^.

The oxidized structure was solved by the molecular replacement method with the CNS program [22] using the structure of HiPIP in the reduced state at 100 K (PDB-ID: 3A39 [17]) as an initial model. One HiPIP molecule is contained in the crystallographic asymmetric unit. The initial refinements with isotropic atomic displacement parameters (ADPs) were carried out using the CNS program up to 1.2 Å resolution. The structure was monitored and corrected with the COOT program [23] according to the 2*F*_obs_-*F*_calc_ and *F*_obs_-*F*_calc_ maps. Further refinements were performed with the SHELXL program [24] with anisotropic ADPs. Restraints on bond lengths and angles were removed for all single conformation residues. Positions of hydrogen atoms were refined as a riding model, while those for some hydrogen atoms that were largely out of the electron density region were manually corrected. The bulk solvent correction was applied with the SWAT option. The *R*_work_ and *R*_free_ factors in the resolution range from 15 Å to 0.8 Å were 11.6% and 12.9%, respectively. The structure for the reduced HiPIP at 40 K was also determined in the same way. Refinement statistics for both states are listed in Table 2. The anisotropy, which is the ratio of the smallest to the largest eigenvalue of the ADPs, was calculated with the PARVATI program [25] in order to compare difference in the thermal fluctuation in detail. The refined structures were validated with the MolProbity program [26]. Figures for molecular models were prepared using the PyMol program [27]. The coordinates and structural factors for the oxidized and reduced crystals have been deposited in the Protein Data Bank under accession numbers 5WQQ and 5WQR, respectively.

**Table 2:** Refinement statistics.

Data set	Oxidized	Reduced
Resolution range (Å)	15–0.8	15–0.8
*R*_work_^a^ / *R*_free_^b^ (%)	11.6/12.9	10.6/11.8
No. of non-hydrogen atoms		
Protein	695	680
Fe-S cluster	8	8
Sulfate/Glycerol	35/18	35/18
Water oxygen	160	149
No. of hydrogen atoms		
Hydrogen of the protein	673	651
Hydrogen of waters	34	31
Average temperature factor (Å^2^)	5.0	6.0
Protein	3.8	4.3
Fe-S cluster	1.9	2.0
Sulfate/Glycerol	10.4	10.4
Water	14.7	13.5
Mean anisotropy^c^		
Protein	0.40	0.45
Fe-S cluster	0.60	0.69
Sulfate/Glycerol	0.21	0.26
Water	0.29	0.30
Multi-conformational residues	13	11
Ramachandran plot^d^ (%)	98.9/1.1/0	97.8/2.2/0

^a^ *R*_work_ = Σ_hkl_||*F*_obs_|−|*F*_calc_||/Σ_hkl_|*F*_obs_|.

^b^ *R*_free_ was calculated by using the 5% of the reflections that were not included in the refinement as a test set. During the refinement, Bijvoet pairs were treated as different reflections.

^c^ Anisotropy is defined as the ratio of the smallest to the largest eigenvalue of the anisotropic displacement parameter matrix.

^d^ Favored/Allowed/Outliers.

## Results and discussion

### Verification of the redox states

**Fig 1:**
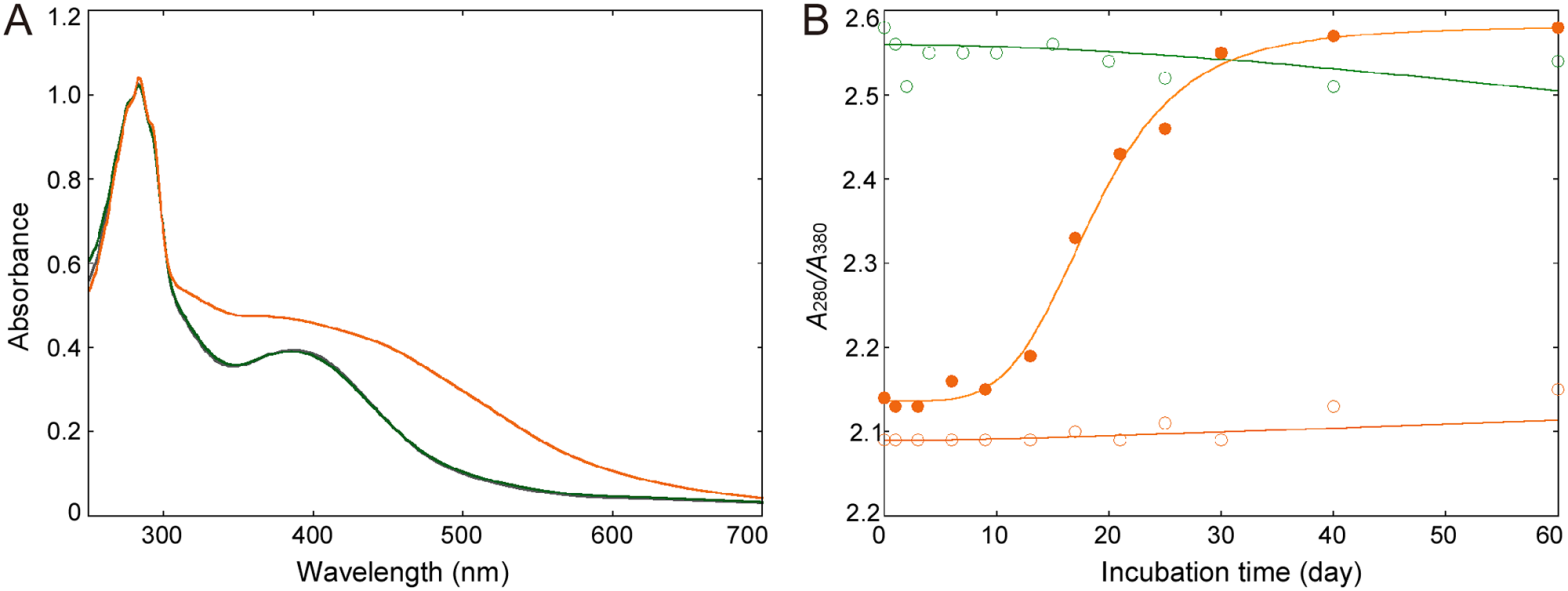
Redox changes of HiPIP in the solution. (A) UV-visible spectra for the oxidized and reduced states are indicated as orange and green lines, respectively. In addition, the spectrum for the non-treated sample is indicated as a gray line, while it is overlapped with the green line. The spectra were scaled by fitting to the absorbance at 280 nm. (B) Changes in the ratio *A*_280_/*A*_380_ as a function of incubation time. Green circles: reduced HiPIP in 100 mM sodium citrate (pH 4.5); orange filled circles: oxidized HiPIP in 100 mM sodium citrate (pH 4.5); orange circles: oxidized HiPIP in 1.0 M ammonium sulfate, 100 mM sodium citrate (pH 4.5) and 2 mM K_3_[Fe(CN)_6_].

The oxidized form of HiPIP was prepared by treatment with the oxidizing agent K_3_[Fe(CN)_6_]. The UV-visible spectra indicate that the *A*_280_/*A*_380_ for the oxidized form was 2.1, while that for the reduced form was 2.5 (Fig 1A). The value for the oxidized form was changed during long incubation at room temperature (Fig 1B). On the other hand, the reduced form exhibited only small spectral changes in which one-seventh of the reduced HiPIP is oxidized during incubation for two months, despite the absence of reducing agent. The change of the oxidized form could be prevented by the addition of ammonium sulfate and K_3_[Fe(CN)_6_], which were used in the crystallization solution. These observations indicate that the preparation was successful and that the oxidized state of HiPIP can be retained during the crystallization process.

### Crystallographic data analysis

**Fig 2:**
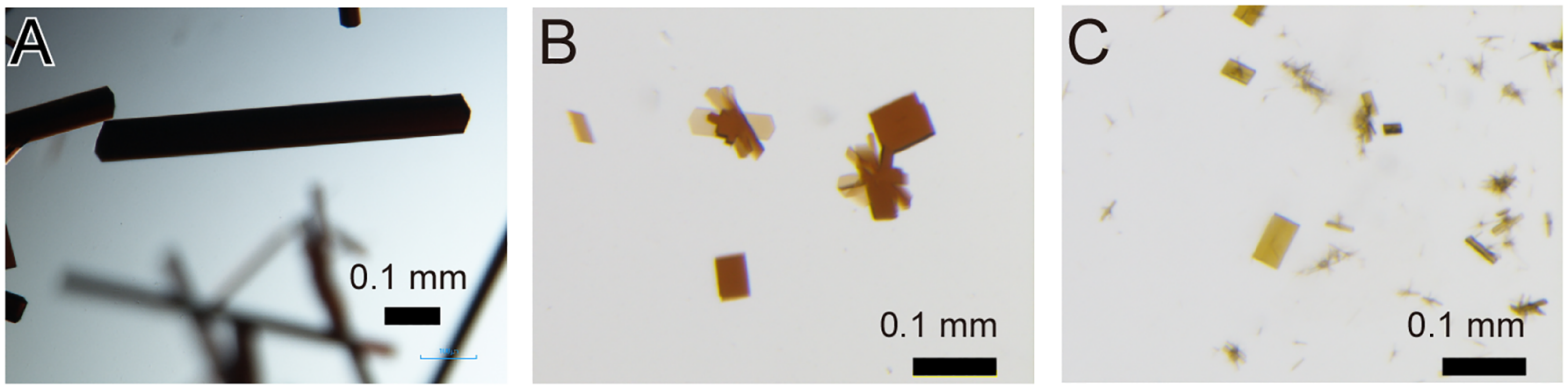
Crystals of HiPIP. (A) Photograph of crystals for oxidized HiPIP. The scale bars indicate 0.1 mm. (B) Small and thin crystals of oxidized HiPIP are shown in order to distinguish crystal colors of oxidized HiPIP from those of reduced HiPIP in panel c. (C) Photograph of small and thin crystals of reduced HiPIP.

Structural changes come from oxidation can be restricted or altered by crystal packing, if the reduced HiPIP is oxidized in the crystal. Therefore, crystals of the oxidized state were produced from the oxidized HiPIP in the solution in order to elucidate the true structural differences accompanying the redox changes. Crystals of the oxidized form were obtained under conditions almost identical to those used for the reduced form. The crystals had a plate-like shape with the typical maximum length of around 0.5–1.0 mm (Fig 2A). The crystals were colored almost black because of the high optical density. The color for small and thin crystals was slightly different from that for the reduced form (Fig 2B and 2C). Crystals with dimensions of ~1.0×0.2×0.1 mm^3^ were used for the data collections. The maximum dose for the data sets was estimated to be 7×10^5^ Gy, which was two orders of magnitude smaller than the recommended dose limits (2−3×10^7^ Gy) [28,29]. In addition, diffraction data sets were collected at 40 K with a helium stream. It has been reported that the photoreduction of metalloproteins can be inhibited significantly at 10–40 K [30,31]. Therefore, we considered that the damage was sufficiently negligible for elucidation of the redox-coupled structural changes. Although the space group and cell constants are identical to those for the reduced HiPIP crystals, the diffraction data show a relatively low isomorphism with the *R*_iso_ value of ~16% (Table 1). These crystallographic properties imply that small but significant changes occur at the central iron-sulfur cluster in which atomic scattering factors for iron atoms are much larger than those for atoms comprising the environmental peptide portion.

**Fig 3:**
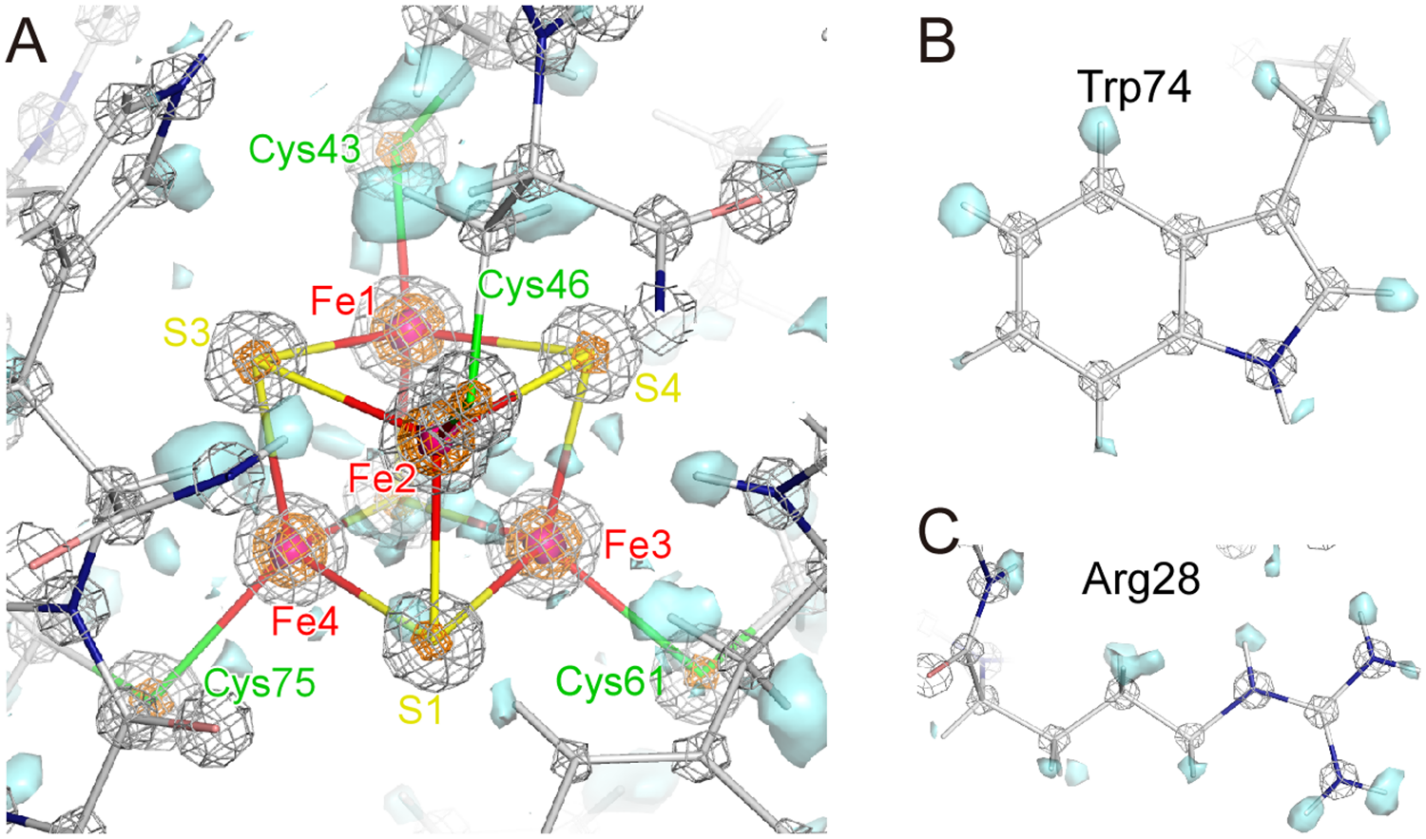
Electron density maps of HiPIP in the oxidized state at 0.8 Å resolution. (A) The electron density map around the iron-sulfur cluster of the oxidized HiPIP at 0.8 Å resolution. The hydrogen omit *F*_obs_−*F*_calc_ map is shown in cyan surface at a contour level of 3σ. The 2*F*_obs_−*F*_calc_ map is shown at contour levels of 5σ (gray mesh), 25σ (orange mesh) and 45σ (magenta surface). (B) The electron density map around Trp74. (C) The electron density map around Arg28.

The electron densities for atoms in the Fe_4_S_4_ cluster are completely separate from each other (Fig 3A). In addition, hydrogen atoms are clearly observed in the omit map for almost all side chains in addition to the main chain (Fig 3B and 3C). The refined structure of the oxidized HiPIP contains all 83 residues, one Fe_4_S_4_ cluster, three glycerols, five sulfate ions and 160 water molecules (Table 2). Multiple (double) conformations were observed for 13 residues. The model contains hydrogen atoms of all residues with the exceptions of some side chain atoms with multi-conformations. 34 hydrogen atoms of water molecules were also included in the refinement calculations. The five sulfate ions are observed in the vicinity of the positively charged residues. No ferricyanide ions are observed in the electron density map.

### Structural comparison with the reduced form

**Fig 4:**
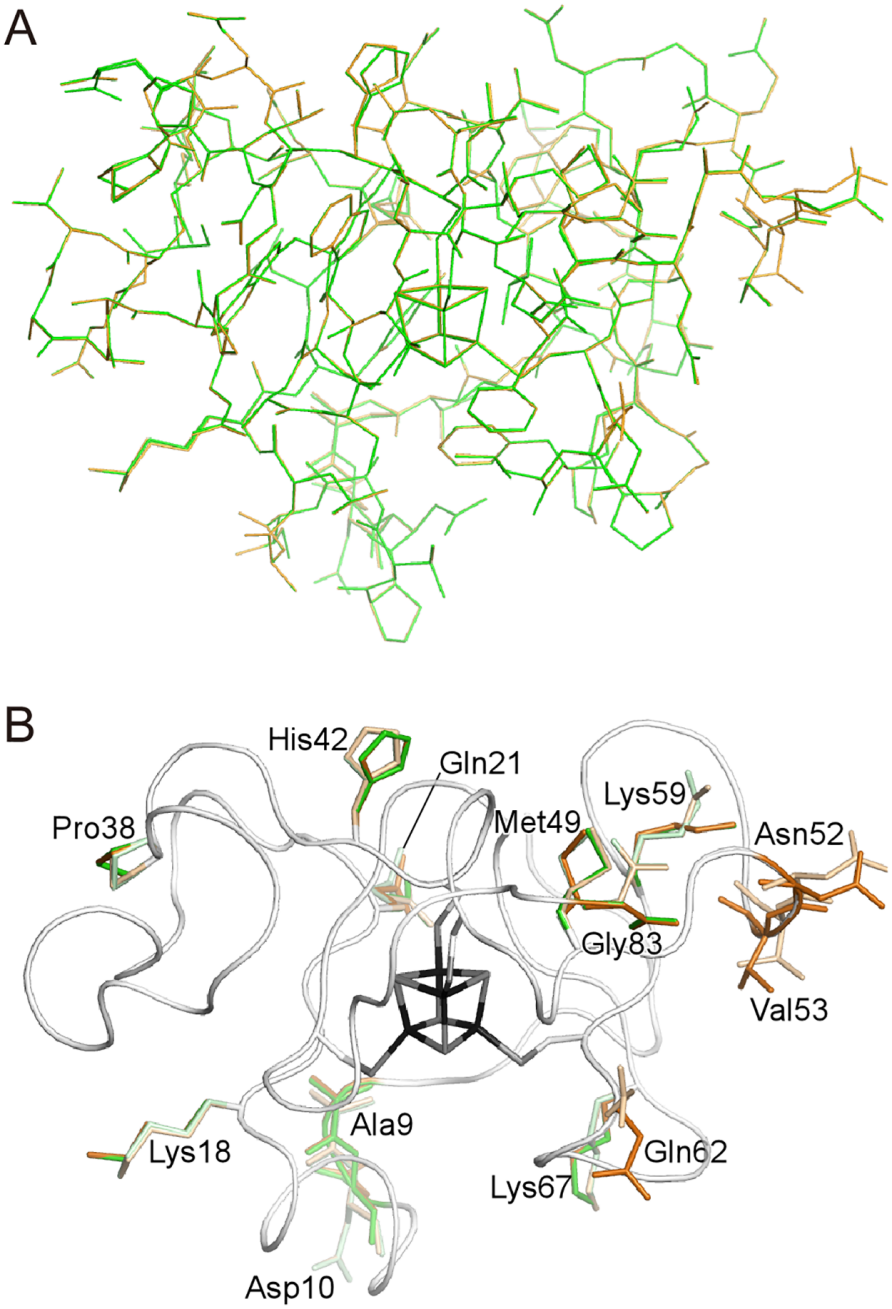
Crystal structures of HiPIP at 0.8 Å. (A) Differences between the reduced and oxidized forms. The structures in the oxidized and reduced states are superimposed and represented in orange and green, respectively. Hydrogen atoms are omitted from the figure for clarity. (B) Multi-conformational residues in the oxidized and reduced states are represented as orange and green sticks, respectively. Depth of colors reflects the occupancy of each conformation at the residue. Single-conformational residues and the iron-sulfur cluster are represented as gray tubes and sticks, respectively.

**Fig 5:**
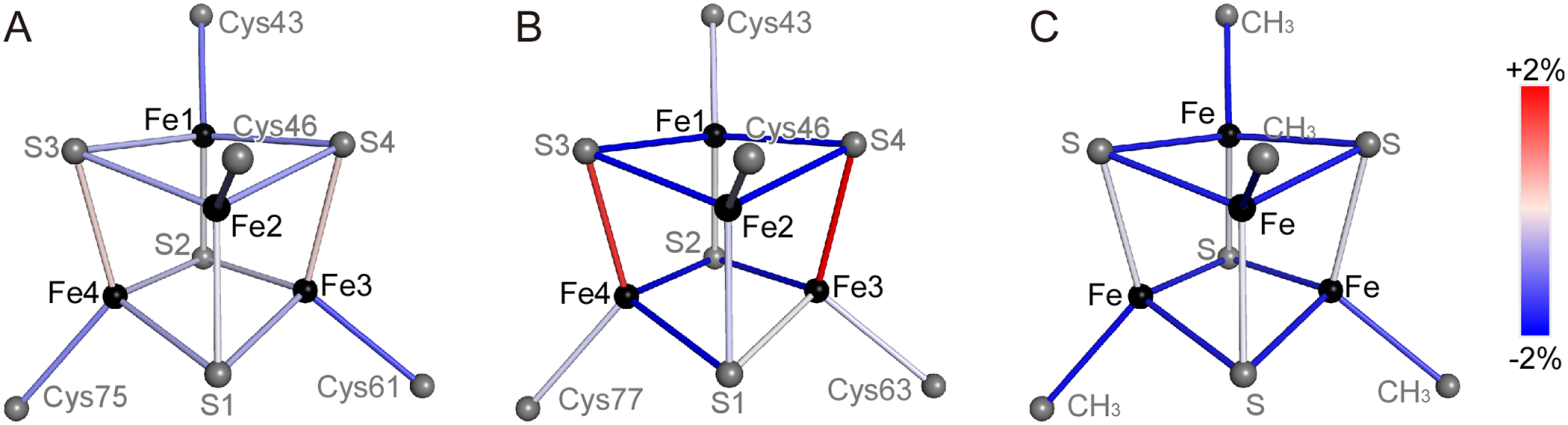
Differences on the respective bonds of the iron sulfur cluster. Bond sticks are colored in red (positive) or blue (negative) according to the difference values in the bond distance, (*d*^Ox^—*d*^Red^)/*d*^Red^. (A) Values are from X-ray analyses of HiPIP from *T*. *tepidum* (this work). (B) X-ray analyses of HiPIP from *A*. *vinosum* [12]. Fe-(Cys-S_γ_) distances are the averaged value of four bonds. (C) Theoretical calculations for [Fe_4_S_4_(SCH_3_)_4_] [33].

No apparent conformational changes between the oxidized and reduced states are detected in the superimposition of the oxidized and reduced structures (Fig 4A). Differences are observed only in multi-conformational residues at the loop region (Fig 4B). This indicates that the differences are not intrinsic due to low accuracies of coordinates. The average root mean square deviations (rmsd) for all the polypeptide atoms and for the main chain atoms are 0.448 and 0.039 Å, respectively. The rmsd value for the iron-sulfur cluster alone is 0.020 Å. This value is significantly larger than the uncertainty of the refinement calculations of 0.003 Å, as estimated by full-matrix least-square refinement (Table 3). Therefore, our results indicated that the two forms actually had slightly different bond lengths at the Fe_4_S_4_ cluster. The Fe_4_S_4_ cluster consists of two Fe_2_S_2_ subclusters, Fe1-S4-Fe2-S3 and Fe3-S1-Fe4-S2 [32]. The intra-subcluster bond lengths are decreased by the oxidation, while inter-subcluster bond lengths are not (Fig 5A). The largest changes are observed in the Fe−S bonds around Fe1 and Fe2. In addition, the bond lengths of Fe- (Cys-S_γ_) for all Fe atoms are also decreased by the oxidation. The almost identical redox-related structural changes are observed for the comparison to the reduced structure at 0.48 Å, which were refined with the multipolar atomic model [18]. The previous X-ray crystallographic analysis of HiPIP from *A*. *vinosum* shows a significantly different magnitude of changes, especially for the expanding Fe3–S4 and Fe4–S3 bonds (Fig 5B) [12]. However, the changes in HiPIP from *T*. *tepidum* are similar to that predicted by theoretical calculation for a model compound [33] (Fig 5C). Our results for the difference in the Fe-S lengths implies that the subcluster composed of Fe1, Fe2, S3, and S4 is important for storing electronic charges in HiPIP. This implication is consistent with the previous results such as charge-density analysis [18] and X-ray absorption spectroscopic analysis [32]. In addition, most distances between the sulfur atoms of the iron-sulfur cluster and the protein environment are elongated upon the oxidation (Table 4). This indicates that the structure of the peptide portion does not follow the contraction of the iron-sulfur cluster.

**Table 3:** Bond length for the Fe_4_S_4_(Cys-S_γ_)_4_ cluster.

	Oxidized	Reduced
FE1-S2	2.215(2)	2.219(2)
FE1-S4	2.269(2)	2.298(2)
FE1-S3	2.297(3)	2.315(2)
FE2-S1	2.208(2)	2.211(2)
FE2-S3	2.277(2)	2.299(2)
FE2-S4	2.286(3)	2.310(3)
FE3-S4	2.249(2)	2.240(2)
FE3-S2	2.296(3)	2.308(2)
FE3-S1	2.288(3)	2.306(3)
FE4-S3	2.270(2)	2.262(2)
FE4-S1	2.271(2)	2.291(2)
FE4-S2	2.283(3)	2.297(2)
FE1-(Cys43-S_γ_)	2.207(2)	2.243(2)
FE2-(Cys46-S_γ_)	2.237(3)	2.263(2)
FE3-(Cys61-S_γ_)	2.221(3)	2.252(2)
FE4-(Cys75-S_γ_)	2.234(2)	2.265(2)

Values in parentheses are estimated standard deviations of the full-matrix least-square refinement.

**Table 4:** Distance between S atoms of the cluster and the peptide portion.

	Oxidized	Reduced
(Tyr19-C_δ1_)-S2	3.673(7)	3.662(7)
(Phe48-N)-(Cys46-S_γ_)	3.494(6)	3.448(5)
(Phe48-C_δ2_)-S1	3.830(9)	3.812(8)
(Leu63-N)-(Cys61-S_γ_)	3.464(6)	3.447(5)
(Leu63-C_δ1_)-S1	3.556(8)	3.544(7)
(Phe64-C_δ2_)-(Cys61-S_γ_)	3.738(8)	3.708(7)
(Phe64-C_ε2_)-S2	4.028(8)	3.988(7)
(Ile69-C_γ2_)-S2	3.938(8)	3.913(7)
(Asn70-O)-(Cys43-S_γ_)	3.310(5)	3.351(5)
(Cys75-N)-S3	3.419(6)	3.398(6)
(Ser77-N)-(Cys75-S_γ_)	3.435(6)	3.380(6)
(Trp78-C_δ1_)-S3	3.813(7)	3.776(6)
(Thr79-N)-(Cys46-S_γ_)	3.572(6)	3.523(6)

Values in parentheses are estimated standard deviations of the full-matrix least-square refinement.

**Fig 6:**
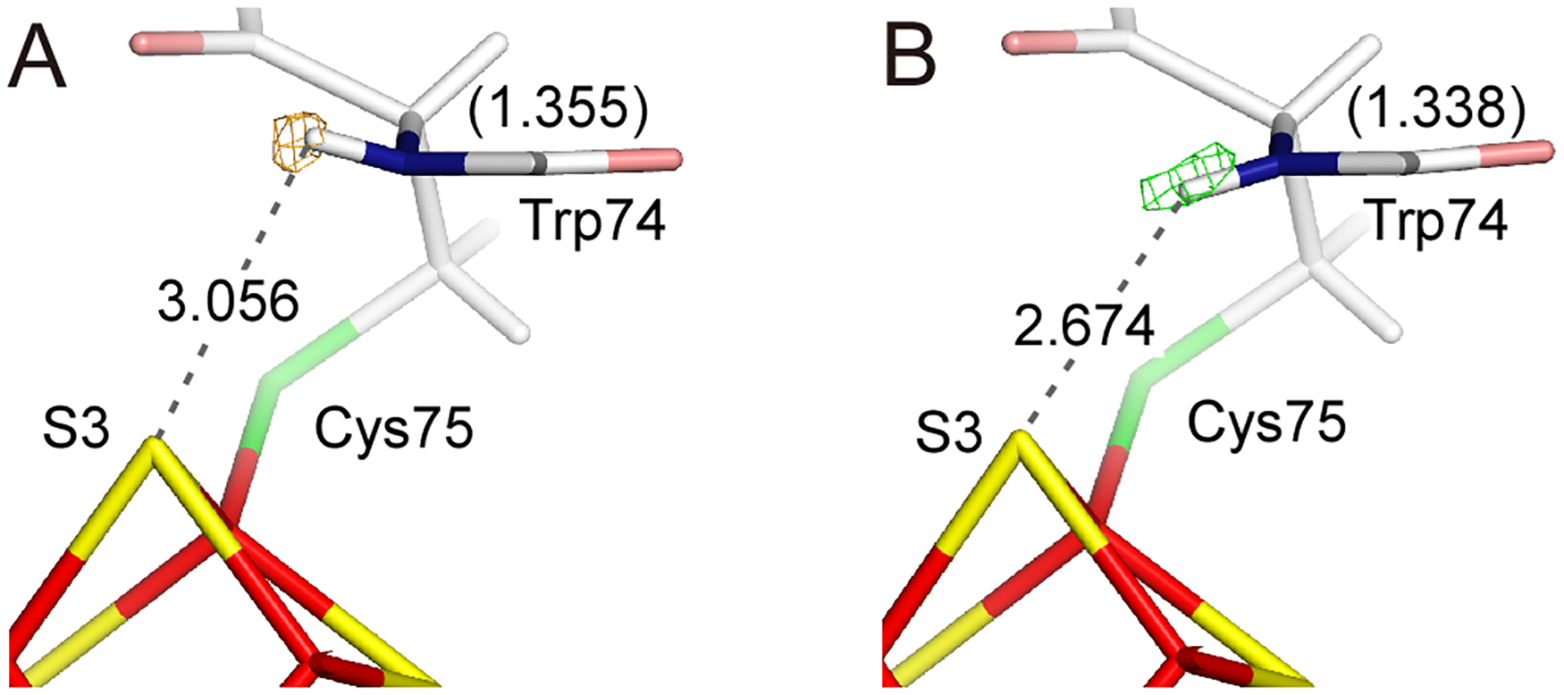
Positional changes of the amide-hydrogen atom of Cys75 on the redox change. (A) The hydrogen omit *F*_obs_−*F*_calc_ map for the oxidized state is shown as an orange mesh at a contour level of 4σ. The value without parentheses is the bond length between amide-H of Cys75 and S3, while that with parentheses is the bond length for the C-N bond. (B) The hydrogen omit *F*_obs_-*F*_calc_ map for the reduced state is shown as a green mesh at a contour level of 4σ.

Although electron densities for the hydrogen atoms were observed in the hydrogen omit map, the almost all of the positions were only refined as riding atoms. Therefore, the positions can not be compared accurately. However, some hydrogen atoms show significant differences between the two states. The amide-H of Cys75, which interacts with the S3 atom of the iron-sulfur cluster, is located outside the normal position in the oxidized state, but not in the reduced state (Fig 6). The hydrogen-bonding distances between the amide-H and S3 in the oxidized and reduced states are 3.056 Å and 2.674 Å, respectively. Moreover, a significant difference of ~0.02 Å between the two states is observed for the lengths of the peptide bond between Trp74 and Cys75 (Fig 6). This may have been linked to the planarity of the peptide bond, which defines the delocalization degree of the valence electrons [34].

**Fig 7:**
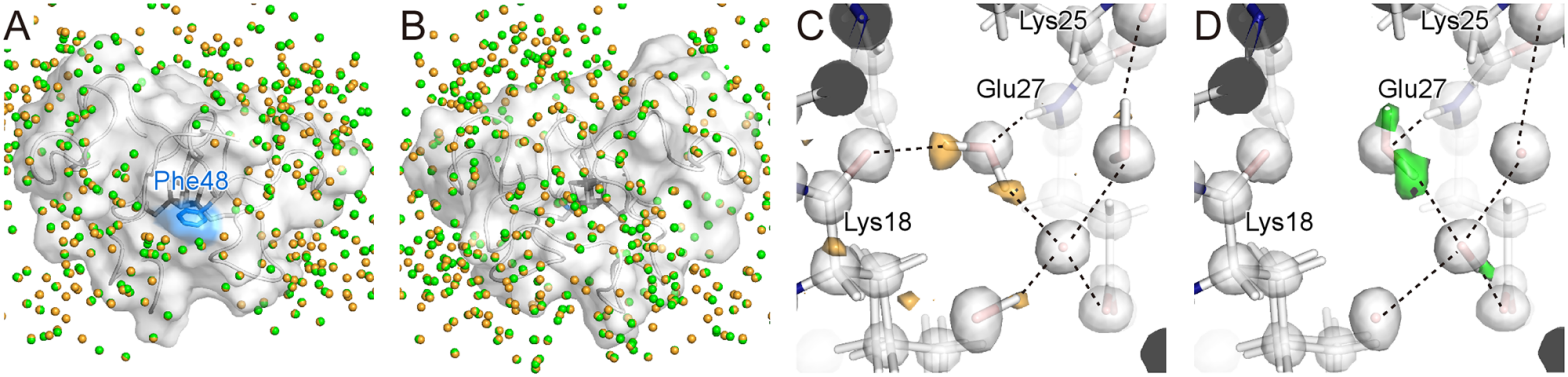
Water molecules on the surface of HiPIP. (A) Oxygen atoms of waters for the oxidized and reduced HiPIP are colored in orange and green, respectively. HiPIP is represented as a transparent surface model. The side chain of Phe48 on the putative electron-transfer pathway [36] is colored in blue. (B) The backside surface. (C) A water cluster on the surface of HiPIP in the oxidized state. The hydrogen omit *F*_obs_-*F*_calc_ map is shown in orange surface at a contour level of 3σ. The 2*F*_obs_-*F*_calc_ map is shown in gray surface at contour levels of 2σ. (D) The same water cluster in the reduced state. The hydrogen omit *F*_obs_-*F*_calc_ map is shown in green surface at a contour level of 3σ.

Many water molecules are observed on the molecular surface of HiPIP, while the putative interaction side with RC around Phe48 binds no waters due to covering by a neighbor molecule in the crystal packing (Fig 7A). None of the water molecules exhibit significant changes in position or anisotropy of ADP between the two states (Fig 7A and 7B). This may be due to the small structural changes even on the protein surface. Small structural changes for both the central cluster and molecular surface imply that the reorganization energy of HiPIP at the redox change is very low. This interpretation is consistent with the theoretical calculation for model iron-sulfur clusters [35]. However, the orientations of some water molecules were different (Fig 7C and 7D). The orientations may reflect the direction of the electric field on the protein surface, which are mainly generated from charges of the iron-sulfur cluster. Therefore, we can presume that the redox changes at the central cluster bring the difference of the electrostatic potential to the molecular surface of the interaction site with RC.

## Conclusions

We established a procedure for preparing the high quality crystals of the HiPIP in an oxidized state, and performed a detailed structural determination at 0.8 Å resolution. However, the differences in atomic positions between the oxidized and reduced HiPIP are smaller than previous results with X-ray crystallography and NMR spectroscopy for HiPIP from *A*. *vinosum*, while HiPIP from the bacterium shows a high sequence homology (~90%) with HiPIP from *T*. *tepidum*. More detailed investigations focused on the distributions of the valence electrons and atomic charges around the iron-sulfur cluster will be crucial to reveal the electronic structural changes of the protein. Our preparation method will allow further crystallographic analyses to resolve these questions.
